# Pain levels and patient comfort after lower limb arthroplasty comparing i.v. patient-controlled analgesia, continuous peripheral nerve block and neuraxial analgesia: a retrospective cohort analysis of clinical routine data

**DOI:** 10.1186/s13018-022-03277-0

**Published:** 2022-08-12

**Authors:** Alina Yurutkina, Sven Klaschik, Pascal Kowark, Annette Gass, Carolina Link, Thomas Martin Randau, Jorge Jiménez-Cruz, Mark Coburn, Tobias Hilbert

**Affiliations:** 1 Department of Anesthesiology and Intensive Care Medicine, University Hospital Bonn, Venusberg-Campus 1, 53127 Bonn, Germany; 2 Department of Orthopedics and Trauma Surgery, University Hospital Bonn, Bonn, Germany; 3 Department of Obstetrics and Prenatal Medicine, University Hospital Bonn, Bonn, Germany

**Keywords:** Hip arthroplasty, Knee arthroplasty, Peripheral nerve block, Patient-controlled analgesia

## Abstract

**Background:**

Insufficient pain control after lower limb arthroplasty results in delayed recovery and increased risk for pain chronicization. The ideal kind of analgesia is still discussed controversially. We conducted a retrospective analysis of single-center routine data from a German university hospital, including patients receiving either total hip (THA) or knee arthroplasty (TKA).

**Methods:**

All patients received general anesthesia. Patients undergoing THA received either continuous epidural ropivacaine infusion (0.133%, Epi) or patient-controlled analgesia (PCA) with the Wurzburg Pain Drip (tramadol, metamizole and droperidol, WPD) or with piritramide (Pir). After TKA, patients received either continuous femoral nerve block (ropivacaine 0.2%, PNB) or Pir.

**Results:**

The analyzed cohort comprised 769 cases. Use of WPD after THA (*n* = 333) resulted in significantly reduced Numeric Rating Scale (NRS) values at rest, compared to Epi (*n* = 48) and Pir (*n* = 72) (.75 [IQR 1.14] vs. 1.17 [1.5], *p* = .02 vs. 1.47 [1.33], *p* < .0001) as well as maximum NRS scores (2.4 [1.7] vs. 3.29 [1.94], *p* < .001 vs. 3.32 [1.76], *p* < .0001). Positive feedback during follow-up visits was significantly increased in patients with a WPD PCA (*p* < .0001), while negative feedback (senso-motoric weakness/technical problems/nausea/dizziness/constipation) was particularly increased in Epi patients and lowest in those with WPD (*p* < .0001). After TKA, Pir (*n* = 131) resulted in significantly reduced NRS values at rest, compared to PNB (*n* = 185) (1.4 [1.4] vs. 1.6 [1.68], *p* = .02). Positive feedback was increased in patients with a Pir PCA in comparison with PNB (*p* = .04), while negative feedback was increased in PNB patients (*p* = .04). Overall, WPD presented with the lowest rate of any complications (8.7%), followed by Pir (20.2%), PNB (27.6%) and Epi (31.3%) (*p* < .001).

**Conclusions:**

In the assessed population, the use of a WPD PCA after THA offered better pain control and patient comfort in comparison with continuous epidural or piritramide-based analgesia. After TKA, the use of a Pir PCA provided superior analgesia and a lower complication rate compared to continuous PNB.

## Background

Lower limb arthroplasty (LLA) usually improves patients’ quality of life to a great extent, but is, on the other hand, associated with high-level postoperative pain [1]. Poor pain control after LLA results in delayed recovery and hospital discharge [2]. In addition, to achieve optimal postoperative functional outcomes, intensive physiotherapy is required for both the operated and the contralateral side, likewise requiring effective analgesia [1]. Less invasive surgical techniques to improve outcomes after LLA have been developed over the past years, but persistent and chronic pain may still complicate an otherwise successful procedure [3]. Wylde et al. demonstrated persistent postsurgical pain (PPSP) in patients after total hip (THA) or total knee arthroplasty (TKA) in up to 44%, with severe to extreme pain reported by up to 15% of the patients [3]. No doubt that this adds significant further costs for the healthcare systems worldwide. Therefore, optimal postoperative analgesia is mandatory to prevent chronicization of pain following LLA [4].

However, the ideal kind of analgesia is still discussed controversially. This is not least due to the fact that different and even contradictory demands are made on an optimal postoperative pain therapy: high analgesic efficacy without motor impairment and limitation of patient mobilization, good tolerability without dizziness, constipation or nausea, and an application controlled by the patient and adapted to his or her needs. In their review, Højer Karlsen et al. concluded that “[…] *the available randomized placebo-controlled trials* [do] *not allow a designation of a ‘best proven intervention’*” [5].

We analyzed single-center routine data from a German university hospital, with more than 750 patients who received either hip or knee arthroplasty, to gain further insights into this topic. Records from the in-house acute pain service (APS) from the years 2016 and 2018 were evaluated to identify differences in pain control and patient comfort between epidural analgesia (Epi), continuous peripheral nerve block (PNB) and i.v. patient-controlled analgesia (PCA).

## Methods

All analyses were performed in accordance with the Declaration of Helsinki. The local ethics committee (University Hospital Bonn, Germany) considered the study to be compliant with the terms of the current professional codes and regulations and thereby approved the study protocol. Due to its retrospective character, written informed consent was waived.

Protocols from the in-house APS from 2016 and 2018 were retrospectively evaluated. All patients aged over 18 years undergoing orthopedic lower limb surgery were screened for eligibility. Patients having received surgery other than primary or revision hip or knee arthroplasty were excluded. In addition, patients without the need for advanced postoperative pain therapy (continuous neuraxial analgesia or continuous peripheral nerve block or i.v. PCA) were excluded.

All patients received general anesthesia. THA was performed via the modified direct lateral approach according to Bauer/Hardinge. Patients received either continuous epidural ropivacaine infusion (0.133%, Epi) or PCA with the Wurzburg Pain Drip (tramadol, metamizole and droperidol, continuous and bolus application [15 mg/h tramadol, bolus 10 mg, 10-min lockout interval]), WPD) or with piritramide (bolus application only [2 mg, 8-min lockout interval], Pir). Epidural catheters were placed before inducing general anesthesia but were not used intraoperatively. After surgery, ropivacaine (0.2%) was administered, and a CADD™-Solis pump (Minneapolis, Minnesota, US) filled with ropivacaine (0.133%) was connected to the catheter. The pump infuses local anesthetic continuously (6 ml/h). For TKA, patients received either continuous femoral nerve block (ropivacaine 0.2%, PNB) or Pir PCA. Peripheral nerve block catheters were placed after inducing anesthesia and before surgery using ultrasound, and single-shot ropivacaine (0.375%) was administered via the catheter. After surgery, a CADD™-Solis pump filled with ropivacaine (0.2%) was connected to the catheter (continuous infusion 6 ml/h, bolus injection 4 ml, 60-min lockout interval).

The mode of postoperative pain therapy was chosen according to our in-hospital standard operating procedure. This changed during the observation period between 2016 and 2018 from continuous Epi and from Pir PCA to WPD PCA as the preferred therapy for THA patients and from continuous PNB to Pir PCA for TKA patients, allowing for comparison between the different groups. In some cases, i.v. analgesia was combined with additional preoperative single-shot fascia iliaca (THA patients) or adductor canal block (TKA patients) with ropivacaine 0.375% without placing a catheter or with local infiltration analgesia of the hip or knee joint administered by the surgeon at the end of the surgery, respectively. Patients were prescribed a rescue medication with nonsteroidal anti-inflammatory drugs (NSAIDs) or opioids according to the WHO analgesic ladder. The basal infusion rates of the pumps as well as the dosage of bolus injections were individually adjusted according to the Numeric Rating Scale (NRS, ranging from 0 [no pain] to 10 [strongest pain]) values reported by the patients during the APS follow-up visits.

The quality of postoperative analgesia was evaluated by routine APS follow-up visits twice a day and was documented using a standardized protocol sheet. Additional irregular visits were carried out if necessary. The following parameters were extracted from the APS protocols and used for intergroup comparison:

- duration of advanced postoperative therapy using i.v. PCA, Epi or PNB (days)
- patient round frequency (total number of [routine and irregular] APS follow-up visits, divided by the duration of advanced postoperative therapy)
- pain level at rest, defined as averaged NRS values (cumulative NRS scores, divided by round frequency)
- maximum pain level during mobilization and physiotherapy sessions, defined as averaged NRS values (cumulative NRS scores, divided by round frequency)
- frequency of bolus requests (total number of bolus requests, divided by the duration)
- bolus ratio (total number of bolus requests, divided by the number of applied boluses)
- frequency of positive feedback (number of APS follow-up visits with positive feedback [the patient is satisfied, no or only moderate pain, no problems with mobilization, no nausea/dizziness/senso-motoric weakness/technical problems handling the pump], divided by the total number of APS visits)
- frequency of negative feedback (number of APS follow-up visits with negative feedback [the patient is dissatisfied, severe pain even at rest, impossible mobilization, nausea/dizziness/senso-motoric weakness/technical problems handling the pump], divided by the total number of APS visits)

Additional parameters analyzed comprised type of surgery performed (primary or revision hip of knee arthroplasty), patient age, gender, height, weight, body mass index, history of chronic analgesic medication, use of additional single-shot regional analgesia before surgery and need for postoperative opioid-based co-analgesia. Furthermore, reported complications were grouped into either systemic (nausea/dizziness/sedation/constipation), senso-motoric or technical problems (catheter dislocation/loss of i.v. access).

Statistical analyses and visualization were performed using MS Excel 2019 (Microsoft Corp., Redmond, CA, USA) and GraphPad PRISM 8 (La Jolla, CA, USA). Shapiro–Wilk test was used to test for normal distribution of values. In case of missing values, mean imputation method was used to deal with it. All data are presented as absolute numbers (with percentage) or as median values with an interquartile range (IQR [Q25–Q75]). Kruskal–Wallis test or Mann–Whitney test was used for intergroup comparison. Differences in history of chronic analgesic medication, the use of additional single-shot regional analgesia before surgery and the need for postoperative opioid-based co-analgesia were calculated using Fisher’s exact test. The alpha level was set to 0.05. All datasets are available from the author on reasonable request.

## Results

**Fig. 1:**
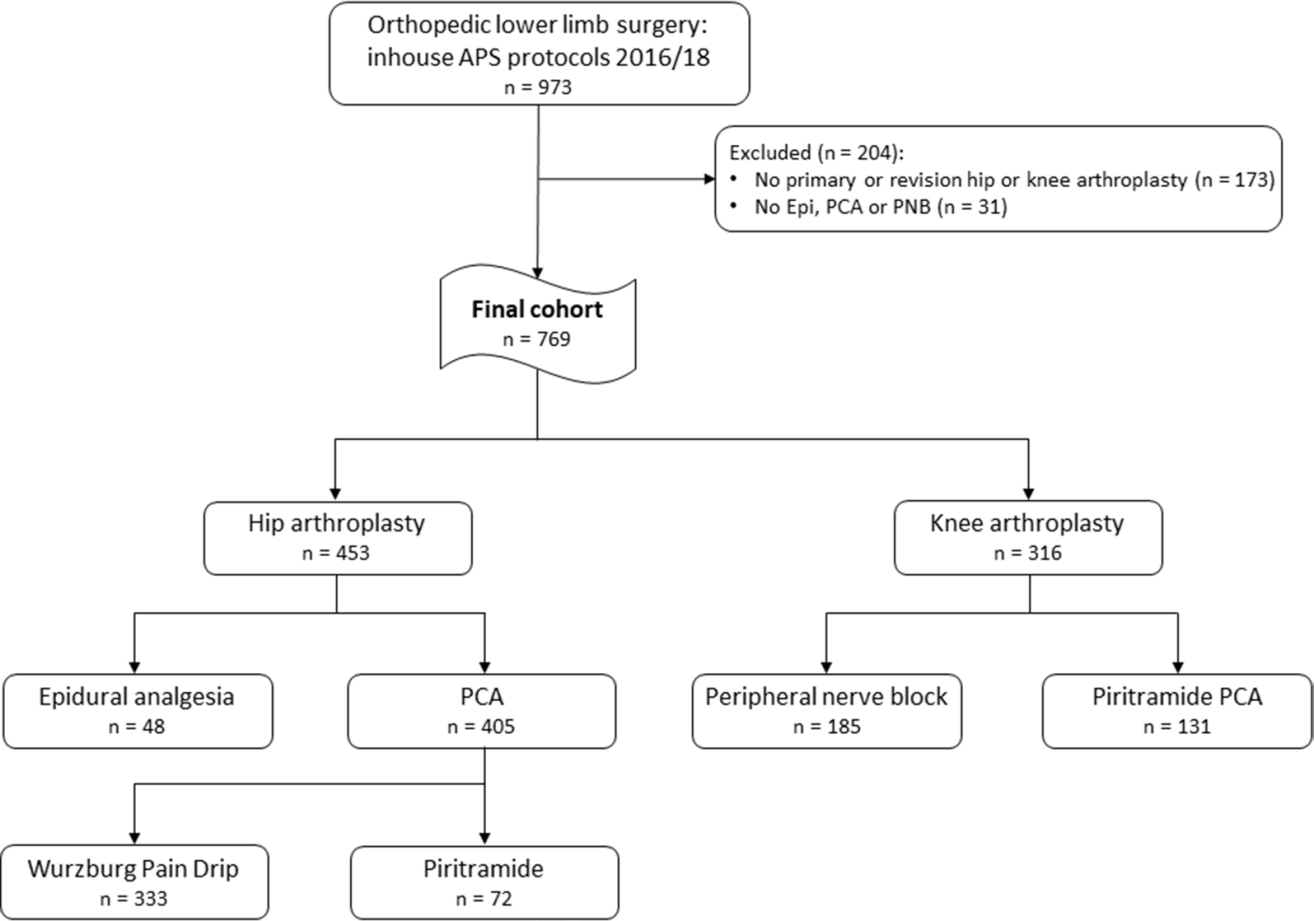
Retrospective study design and patient flowchart. APS: acute pain service, Epi: epidural analgesia, PCA: i.v. patient-controlled analgesia, PNB: continuous peripheral nerve block

In total, 973 APS protocols from patients receiving orthopedic lower limb surgery during the years 2016 and 2018 were identified to be eligible for analysis. Two hundred and four protocols had to be excluded: In 173 cases, surgery other than primary or revision hip or knee arthroplasty had been performed (osteosynthesis, tumor resection, prosthetic lower limb restoration and others), and in 31 cases, patients received neither Epi nor PCA nor PNB (Fig. 1). The final cohort used for analysis comprised 769 cases. Missing values occurred for few of the analyzed parameters, and their frequency was low: height 6, weight 5, BMI 6, positive feedback 1 and negative feedback 1. For the other analyzed parameters, datasets were complete. Hip surgery was performed in 453 cases: Primary prosthetic hip replacement was performed in 263 patients, while an arthroplasty prosthesis was revised in 190 cases (removal, re-installation or direct replacement surgery). In hip surgery patients, analgesia was performed via an epidural catheter in 48 cases (Epi) and via i.v. PCA in 405 cases. Of those, 333 patients received a Wurzburg Pain Drip (WPD), and 72 a piritramide PCA (Pir). Knee surgery was performed in 316 cases: primary prosthetic knee joint replacement (*n* = 145) and TKA revision surgery (*n* = 171). A continuous peripheral nerve block was applied to 185 patients (PNB), and Pir PCA was used in 131 cases. Table 1 provides an overview of the basic patient characteristics. Shapiro–Wilk test revealed non-normally distributed data; thus, a nonparametric analysis was applied.

**Table 1:** Patient characteristics

	Total	THA	Epi	WPD	Pir	TKA	PNB	Pir
Number (n [%])	769	453 [58.9]	48 [10.6]	333 [73.5]	72 [15.9]	316 [41.1]	185 [58.5]	131 [41.5]
Male sex (n [%])	315 [41.0]	202 [44.6]				113 [35.8]		
Age (years)	68 [18]	69 [29]				67 [17]		
Height (cm)	169 [14]	170 [13]				168 [16]		
Weight (kg)	80 [25]	78 [24]				85 [27]		
BMI (kg/m^2^)	27.6 [8.1]	27.0 [7.1]				29.2 [9.5]		
Chronic analgesic medication								
Non-opioid (n [%])			18 [37.5]	171 [51.4]	20 [27.8]		77 [41.6] *	73 [55.7]
Opioid (n [%])			8 [16.7] ***	37 [11.1] ***	38 [52.8]		47 [25.4]	26 [19.8]
Additional single-shot RA (n [%])			– [–]	195 [58.6] *	30 [41.7]		185 [100] ***	74 [56.5]
Need for opioid co-analgesia (n [%])			7 [14.6] ***	13 [3.9] ***	34 [47.2]		53 [28.6]	25 [19.1]

*THA* total hip arthroplasty, *TKA* total knee arthroplasty, *Epi* epidural analgesia, *WPD* Wurzburg Pain Drip patient-controlled analgesia (PCA), *Pir* piritramide PCA, *PNB* continuous peripheral nerve block, *BMI* body mass index, *RA* regional anesthesia

Data are given as absolute numbers (with percentage) or as median (with IQR). Fisher’s exact test. *p < .05, ***p < .005 (vs. Pir)

**Fig. 2:**
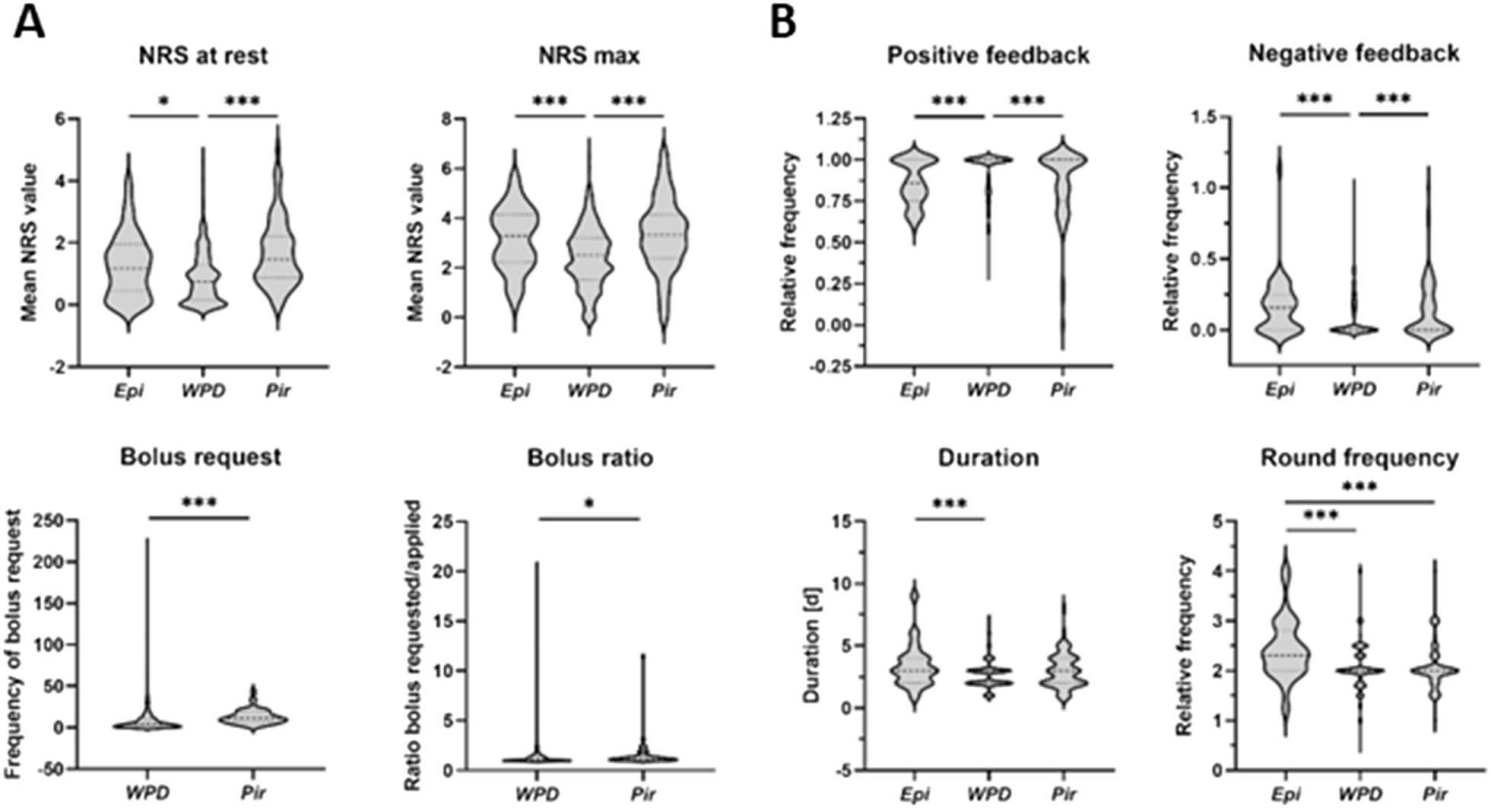
Postoperative analgesia following total hip arthroplasty. Protocols from the in-house acute pain service from 2016 and 2018 were retrospectively evaluated. Data from patients after total hip arthroplasty with either epidural (Epi, *n* = 48) or i.v. patient-controlled analgesia with the Wurzburg Pain Drip (WPD, *n* = 333) or piritramide (Pir, *n* = 72), respectively, are given. The figure shows median average Numeric Rating Scale (NRS) values at rest and maximum NRS values (**A**, upper panels), median number of PCA bolus requests and the median ratio between requested and administered boluses (**A**, lower panels). In **B**, positive and negative feedback during regular follow-up visits (expressed as median relative frequency, upper panels) and median duration and frequency of patient visitations (lower panels) are shown. Data are visualized as violin diagrams with median and interquartile range (25–75), indicated by the dashed lines. Kruskal–Wallis test or Mann–Whitney test was used for comparison. *p < .05, ***p < .005

Figure 2 demonstrates results after a hip surgery. Use of WPD resulted in significantly reduced median averaged NRS values at rest, compared to Epi and Pir (0.75 [IQR 1.14] vs. 1.17 [1.5] [*p* = 0.02] vs. 1.47 [1.33] [*p* < 0.0001]) as well as maximum NRS scores (2.4 [1.7] vs. 3.29 [1.94] [*p* < 0.001] vs. 3.32 [1.76] [*p* < 0.0001]) (Fig. 2A). Comparing the two i.v. PCA procedures, patients with a Pir PCA requested bolus injections significantly more often than WPD patients (11.25 [11.15] vs. 4.0 [8.45] injections per day [*p* < 0.0001]). As shown in Fig. 2B, frequency of positive feedback during APS follow-up visits was significantly increased in patients with a WPD pump in comparison with Epi (1.0 [0.12] vs. 0.86 [0.25] [*p* < 0.0001]), while negative feedback (nausea/dizziness/senso-motoric weakness/technical problems) was particularly increased in Epi patients and lowest in those with WPD (0.16 [0.25] vs. 0.0 [0.13] [*p* < 0.0001]). The overall need for postoperative analgesia was longest when patients had received an epidural catheter (3.0 [2.0] days [*p* = 0.002 vs. WPD]), and those patients further requested significantly more often follow-up visits per day (2.3 [0.8] vs. WPD 2.0 [0.3] [*p* < 0.0001]). No significant differences were found between primary and revision hip surgery in the WPD group (as the mostly used procedure) for any of the parameters analyzed.

**Fig. 3:**
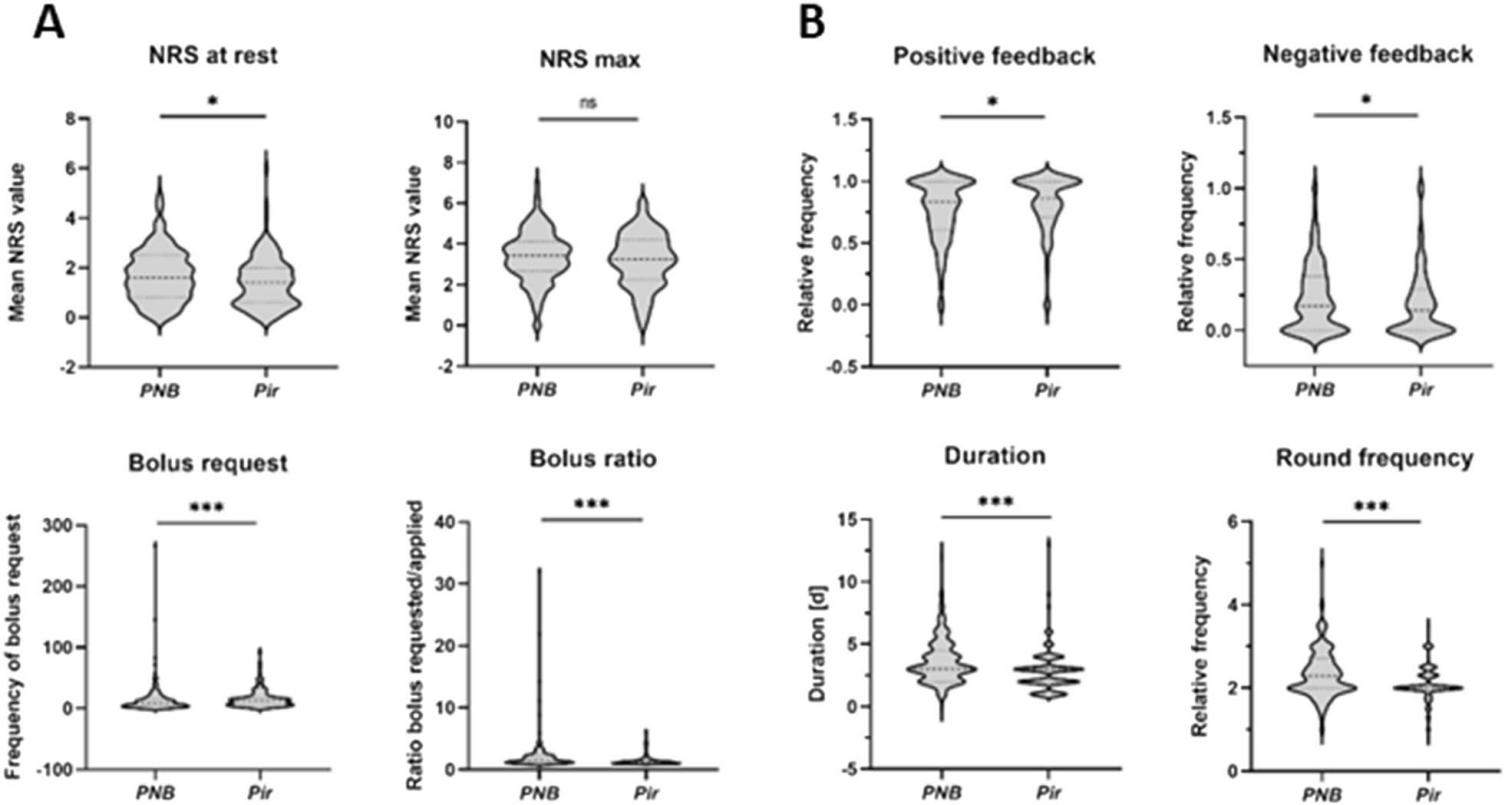
Postoperative analgesia following total knee arthroplasty. Protocols from the in-house acute pain service from 2016 and 2018 were retrospectively evaluated. Data from patients after total knee arthroplasty with either continuous peripheral nerve block (PNB, *n* = 185) or i.v. patient-controlled analgesia with piritramide (Pir, *n* = 131), respectively, are given. The figure shows median average Numeric Rating Scale (NRS) values at rest and maximum NRS values (**A**, upper panels), median number of PCA bolus requests and the median ratio between requested and administered boluses (**A**, lower panels). In **B**, positive and negative feedback during regular follow-up visits (expressed as median relative frequency, upper panels) and median duration and frequency of patient visitations (lower panels) are shown. Data are visualized as violin diagrams with median and interquartile range (25–75), indicated by the dashed lines. Mann–Whitney test was used for comparison. *p < .05, ***p < .005, ns: not significant

Figure 3 shows the results after knee surgery. In those patients, either continuous PNB (femoral nerve block) or Pir PCA was used. Use of Pir resulted in significantly reduced median averaged NRS values at rest, compared to PNB (1.4 [1.4] vs. 1.6 [1.68] [*p* = 0.02]). Maximum NRS scores showed no significant intergroup difference (3.25 [1.95] vs. 3.43 [1.46] [*p* = 0.32]) (Fig. 3A). In both PNB and Pir PCA, patients can (and are encouraged to) request bolus injections (patient-controlled analgesia). Although patients with a Pir PCA often requested bolus injections (13.0 [12.2] vs. PNB 9.0 [12.5] injections per day [*p* = 0.005]), the ratio of requested to administered boluses was lower than in PNB patients (1.2 [0.46] vs. 1.63 [1.07] [*p* < 0.0001]). This is most likely due to the different lockout settings of the pumps (8 min vs. 60 min). As shown in Fig. 3B, there was a slight yet significant increase in frequency of positive feedback during follow-up visits in patients with a Pir pump in comparison with PNB (0.86 [0.29] vs. 0.83 [0.4] [*p* = 0.04]), while negative feedback (senso-motoric weakness/technical problems) was slightly increased in PNB patients (0.17 [0.38] vs. 0.14 [0.29] [*p* = 0.04]). The overall need for postoperative analgesia was longer when patients had received a PNB (3.0 [2.5] days [*p* < 0.0001 vs. Pir]), and those patients further requested significantly more often follow-up visits per day (2.3 [0.7] vs. Pir 2.0 [0.3] [*p* < 0.001]). When comparing primary and revision knee surgery in the PNB group (as the most commonly used analgesic procedure), patients who had received a primary prosthetic knee replacement were less satisfied, giving less frequent positive (*p* = 0.04) and more often negative feedback (*p* = 0.01) during follow-up visits. Moreover, they requested significantly more ropivacaine bolus injections (*p* = 0.007). In contrast, no intergroup differences were observed between primary and revision knee surgery when patients received a Pir PCA instead of a PNB catheter.

Comparison of the subcohort of Pir patients with regard to hip (*n* = 72) or knee surgery (*n* = 131) revealed no differences in any of the observed parameters.

In Table 2, an overview of the reported complications during the use of an individual analgesic procedure is given. Overall, WPD presented with the lowest rate of any complications with reported complications in 29 cases (8.7%), followed by Pir (41 cases [20.2%]), PNB (51 cases [27.6%]) and Epi (15 cases [31.3%]) (Fisher’s exact test *p* < 0.001). While opioid-based i.v. PCA frequently induced systemic complications like nausea, dizziness and constipation, the catheter-based procedures Epi and PNB were most often associated with senso-motoric weakness and technical problems (e.g., catheter dislocation). In some cases, patients did not understand how to properly use the pump.

**Table 2:** Reported complications

	Total	Epi	PNB	WPD	Pir
Number (n [%])	769	48 [6.2]	185 [24.1]	333 [43.3]	203 [26.4]
*Reported complications*					
Systemic (nausea/dizziness/sedation/constipation) (n [%])		1 [2.1]	– [–]	22 [6.6]	31 [15.3]
Senso-motoric weakness (n [%])		10 [20.8]	17 [9.1]	– [–]	– [–]
Technical problems (dislocation/loss of i.v. access) (n [%])		4 [8.3]	34 [18.4]	7 [2.1]	10 [4.9]

*Epi* epidural analgesia, *PNB* continuous peripheral nerve block, *WPD* Wurzburg Pain Drip patient-controlled analgesia (PCA), *Pir* piritramide PCA

Data are given as absolute numbers (with percentage)

Comparison of female (*n* = 454) to male patients (*n* = 315) in the entire cohort regardless of type of surgery or analgesic procedure revealed significantly increased NRS values at rest (1.17 [1.46] vs. 1.0 [1.38] [*p* = 0.001]) as well as maximum NRS scores in female patients (3.0 [1.89] vs. 2.8 [1.84] [*p* = 0.02]). Moreover, women were more dissatisfied with analgesia as they gave positive feedback less often and negative feedback more often than men (*p* = 0.02). The overall need for postoperative analgesia was longer in female patients (*p* = 0.03).

## Discussion

Our results, derived from a retrospective analysis of clinical routine data from a cohort of 769 patients undergoing orthopedic lower limb surgery in a German university hospital, suggest lower resting as well as maximum pain levels and increased patient satisfaction when analgesia after hip arthroplasty was performed using a WPD PCA pump, compared to piritramide PCA or neuraxial analgesia. After knee arthroplasty, the piritramide PCA provided greater patient comfort and best pain control, compared to continuous peripheral nerve block. Overall, i.v. PCA was less often associated with complications, while in epidural and PNB catheters, senso-motoric weakness and technical problems were reported in up to 20% of cases. Furthermore, our results suggest that optimizing postoperative analgesia may be more challenging in female patients.

Both THA and TKA are associated with severe postoperative pain. Optimal functional results and rehabilitation require early mobilization and extensive physiotherapy [1]. Therefore, an effective analgesic therapy in the postoperative period is mandatory. Moreover, this also prevents pain from becoming chronic [3, 4]. Many patients receiving arthroplasty of the lower limb often are accustomed to chronic pain already preoperatively, and chronic use of non-opioids and opioid-based analgesics makes postoperative pain therapy even more challenging [6]. As expected, in our cohort of 769 patients receiving either THA or TKA, 67% of the patients were already taking permanent analgesic medication preoperatively and thus were particularly at risk for further pain chronicization.

In our study, in THA patients, postoperative pain management was achieved by three different and individual possible modalities. Since standard procedures changed during the observation period in our hospital, avoiding neuraxial analgesia after THA, only a small subgroup received an epidural catheter. Although it is reported as being an established and effective way for postoperative pain therapy after THA, recent guidelines do not recommend epidural analgesia due to its undesired specific side effects in LLA (senso-motoric weakness and delayed mobilization) [7]. This is also consistent with our results, as senso-motoric problems occurred in over 20% of the cases, furthermore accompanied by reduced patient satisfaction and increased pain levels and the need for higher follow-up visit frequencies.

In all other THA patients, patient-controlled i.v. analgesia was used, in most cases (73.5%) with a combination of tramadol, metamizole (dipyrone) and droperidol (in Germany referred to as the Wurzburg Pain Drip) and in 15.9% with piritramide. Postoperative analgesia with tramadol in combination with metamizole has repeatedly been shown to be effective; in particular, combining the two compounds elicits significant synergistic analgesic effects [8, 9]. The efficacy of the WPD either as a continuous infusion or as an administration on demand has been demonstrated [10]. Infusion alone requires more interventions to adjust to the individual patient’s needs, and total drug consumption is higher compared to the on-demand administration with no benefit in pain relief, suggesting that PCA may be preferred over continuous infusion [10]. However, in our patients, resting as well as maximum NRS scores were significantly reduced in the WPD group, compared to the Pir PCA (which administers piritramide solely on demand), and WPD patients requested bolus injections only at very low frequencies (4.0 per day). Therefore, this suggests that a combination of a low-dose continuous WPD infusion with the possibility of requesting additional bolus injections, as used in our hospital, seems to be optimal. In our cohort, this offered the best patient comfort and pain relief and the lowest incidence of undesired side effects, compared to the other modalities.

Although slightly less potent, piritramide is a strong opioid like morphine and as such used for postoperative PCA after hip surgery with comparable outcomes [11–13]. In our study, 15.9% of THA patients received Pir PCA. However, our results suggest that the quality of analgesia was considerably worse, compared to WPD, as both resting and maximum NRS scores were highest in the Pir group. In addition, a substantial percentage of patients complained of nausea, dizziness or constipation. This is in line with the results from others and limits patient satisfaction [13]. Of note, in our hospital, standard procedures define that patients with preoperative chronic opioids should receive regional analgesia or Pir PCA, since WPD is usually not sufficient. This results in a higher rate of those patients in the Pir group and may have influenced our findings. Chronic use of opioids obviously makes postoperative pain therapy more challenging [6]. This is in our study likewise reflected by the increased need for additional co-analgesia in hip surgery patients receiving Pir PCA.

Patients after knee surgery received postoperative analgesia either via continuous catheter-based blockade of the femoral nerve or via Pir PCA. Both procedures elicited similar results regarding postoperative pain scores, with Pir PCA resulting in slightly reduced resting NRS values. In 2001, Chelly et al. reported on their results comparing continuous femoral infusion (3-in-1 and sciatic block) with morphine PCA and epidural analgesia after TKA [14]. Interestingly, in contrast to our results, they could demonstrate that PNB provided better analgesia than PCA and even resulted in a reduction in the length of stay in the hospital (which was not investigated in our analysis). Furthermore, similar to our findings, systemic side effects were increased with PCA. Differences in the study designs with a considerably higher infusion rate of local anesthetics (12 ml/h, compared to 6 ml/h with our protocol) may contribute to these different findings.

In our study, senso-motoric weakness and particularly technical problems such as catheter dislocation occurred with continuous PNB in high frequency. The latter resulted in lower patient satisfaction and in an increased need for postoperative supervision. Femoral nerve block (FNB) has long been considered standard postoperative analgesia after knee surgery, however, is associated with quadriceps weakness and risk of fall and therefore delayed mobilization [15]. For this reason, guidelines do not recommend continuous FNB after TKA [16]. Therefore, in our hospital, standard procedures changed during the observation period, and Pir PCA is used in most cases instead of continuous PNB. The latter may be used in exceptional cases, e.g., excessive chronic opioid use or the need for intensified physiotherapy due to pathologic stiffening following arthroplasty. However, a well-organized acute pain service is required to identify and to manage unwanted side effects and technical problems as early as possible, as underlined by the results on PNB described by Chelly et al. Recent data suggest that newly introduced nerve blocks (i.e., quadratus lumborum [QLB] or pericapsular nerve group [PENG] block) potentially motor sparing may be a promising alternative, but evidence from larger-cohort studies is still missing [17].

Although not supported by the data of our retrospective analysis, postoperative pain after TKA is supposed to be greater than following THA. This is in line with our clinical experience and also reported by others [2, 12]. Therefore, our internal standard procedures recommend WPD after hip and Pir PCA after knee surgery. In line with recent guidelines for analgesia following THA and TKA, recommending a multimodal approach for pain management after orthopedic lower limb surgery [7, 16], in our department, both WPD and Pir PCA are usually combined with a preoperative single-shot peripheral nerve block (fascia iliaca block in THA and adductor canal block in TKA) as well as with local infiltration analgesia (LIA) performed by the surgeon at the end of surgery. This has been established during the observation period of our study; therefore, additional single-shot regional anesthesia was not performed in all but mainly in patients of the year 2018. Recent meta-analyses as well as the herein presented results support the benefits of this approach combining long-lasting postoperative analgesia provided by the i.v. PCA with opioid-sparing effects of the PNB while avoiding the disadvantages of catheter-based nerve blockades [18, 19].

Our data suggest more challenging postoperative pain therapy in female compared to male patients, since women complained of more pain than men and were significantly more dissatisfied with pain therapy. Although, from our clinical experience, we did not expect to see such results when analyzing our data, this could also be demonstrated by others. Switon et al. found that pain exacerbation after THA was significantly associated with the female sex [1]. Bonnin et al. demonstrated that women are at a considerable greater risk for residual pain after TKA than men [20]. Therefore, particular attention should be paid to postoperative pain management in female patients following orthopedic lower limb surgery.

Our study has several limitations. Although drawn from a large cohort of more than 750 patients, our results must be interpreted with caution due to the retrospective character of the analysis, posing a risk of selection and performance bias not least due to inadequately balanced groups. The mean imputation method was used to deal with missing data, potentially carrying a risk of bias. Missing values occurred at very low frequencies (max. 0.8% of all collected values per parameter) and for only very few parameters. Moreover, they were missing completely at random. Therefore, although remaining a limitation, mean imputation should not bias estimates and its statistical impact should be negligible [21].

We have no exact data from the preoperative consumption of analgesics; therefore, the impact of chronic pain can only be estimated. Although all data were collected in one single center, surgical technique, as well as the use of additional single-shot regional anesthesia and LIA, was not strictly standardized. Further, our study lacks results on long-term patient outcomes; therefore, we cannot draw any conclusions on pain chronicization and on functional recovery in our cohort. The latter is particularly associated with pain during mobilization and physiotherapy. Moreover, although the length of hospital stay (LOS) was not among the outcome parameters used for intergroup comparison, we must admit that postoperative in-hospital stay in our study seems comparably long, given that today, the average LOS for primary TKA is 0 to 1 day, and patients are usually discharged the same day after primary THA [22]. LOS after a revision surgery is usually between 2 and 4 days. Therefore, our report possibly may not reflect the state of the art with respect to protocols that specifically promote functional recovery and optimize LOS, which might limit the comparability of our results. Last, metamizole and piritramide are not commonly used for postoperative pain therapy in Anglo-American countries, also possibly limiting comparability.

## Conclusions

Taken together, the results of our retrospective analysis of clinical routine data suggest that in the population studied, the use of a WPD PCA after hip arthroplasty provided better pain control and patient comfort in comparison with continuous epidural or piritramide-based analgesia. After knee arthroplasty, the use of a Pir PCA provided superior analgesia and a lower complication rate compared to continuous PNB. In line with our results and according to recent guidelines, a multimodal approach combining single-shot nerve block, local infiltration and long-lasting patient-controlled analgesia should be followed. Particular attention should be paid to pain management in female patients.

## Data Availability

The datasets used and/or analyzed during the current study are available from the corresponding author on reasonable request.

## Abbreviations

LLA: Lower limb arthroplasty
PPSP: Persistent postsurgical pain
THA: Total hip arthroplasty
TKA: Total knee arthroplasty
APS: Acute pain service
Epi: Epidural analgesia
PNB: Peripheral nerve block
PCA: Patient-controlled analgesia
WPD: Wurzburg Pain Drip
Pir: Piritramide
NSAIDs: Nonsteroidal anti-inflammatory drugs
NRS: Numeric Rating Scale
IQR: Interquartile range
FNB: Femoral nerve block
QLB: Quadratus lumborum block
PENG: Pericapsular nerve group block
LIA: Local infiltration analgesia
